# Case report of severe psychiatric sequelae in a 16-year-old female following resection of a purely dopamine-secreting ganglioneuroma

**DOI:** 10.1016/j.ijscr.2019.07.028

**Published:** 2019-07-19

**Authors:** Louis Chai, Sean Ciullo, Rajeev Prasad

**Affiliations:** a St. Christopher’s Hospital for Children, Department of Pediatric Surgery, Philadelphia, PA, 19134, United States; b Drexel University College of Medicine, Hahnemann University Hospital, Department of General Surgery, Philadelphia, PA, 19102, United States

**Keywords:** Ganglioneuroma, Neuroblastic tumors, Hormonally active tumors, Case report

## Abstract

**Background:**

Ganglioneuromas (GN) are rare, benign tumors derived from neural crest cells. They are in the same family of neuroblastic tumors that includes the intermediate ganglioneuroblastoma (GNB) and the malignant neuroblastoma (NB), each of which carries a different prognosis based on tumor histopathology. GNs are generally asymptomatic and usually found incidentally when the tumor becomes palpable or has grown large enough to exert mass effect on adjacent structures. Unlike their malignant counterparts, GNs are rarely hormonally active and usually do not exhibit systemic metabolic activity. We present a case of an adolescent female with a pelvic tumor that was found to be a purely dopamine-secreting GN. Resection resulted in sudden dopamine withdrawal and unexpected severe emotional lability post-operatively.

**Case:**

A 16-year-old female presented with a history of increasingly irregular menses over the past year and was found to have an 8-centimeter pelvic tumor. Subsequent work up revealed the mass to be solely dopamine secreting. The tumor was excised without preoperative hormonal blockade. Post-operatively, the patient developed severe emotional lability and symptoms of depression, likely related to the acute withdrawal of circulating dopamine.

**Conclusion:**

Ganglioneuromas are rarely metabolically active. However, a preoperative endocrine workup should be done to rule out other more commonly hormonally active tumors such as neuroblastomas, pheochromocytomas, and paragangliomas. If isolated dopamine secretion is found, hormonal blockade is not required preoperatively and operative manipulation and removal should be considered safe. However, one should anticipate potential emotional and psychiatric issues post-operatively due to the acute withdrawal of circulating dopamine.

## Highlights

• Ganglioneuromas are rarely hormonally active.
• When metabolically active they typically secrete epinephrine or norepinephrine.
• Exclusively dopamine-secreting tumors are exceedingly rare.
• Surgical excision is the treatment of choice for ganglioneuromas.
• Psychiatric sequelae should be anticipated after resection of a purely dopamine-secreting tumor.

## 1 Introduction

In the pediatric population, neuroblastic tumors are the most common extra-cranial solid tumors and comprise approximately 7–10% of all tumors diagnosed in children [1]. Neuroblastic tumors encompass a spectrum of disease from benign, well differentiated ganglioneuromas (GN) to malignant neuroblastomas (NB) [[2], [3], [4]]. Due to their indolent course, GNs are most commonly diagnosed incidentally when imaging the body for other pathology. GNs may become symptomatic if large enough to be palpated or due to mass effect on surrounding structures. Unlike in NBs, GNs are infrequently hormonally active and therefore, rarely cause symptoms due to the secretion of catecholamines [5,9]. When present however, the hormones typically found to be associated with these symptoms are epinephrine or norepinephrine. We present here a case of a maturing ganglioneuroma with isolated secretion of dopamine that was surgically excised and resulted in emotional lability and symptoms of depression post-operatively. This case has been reported in compliance with SCARE criteria [6].

## 2 Case

**Fig. 1:**
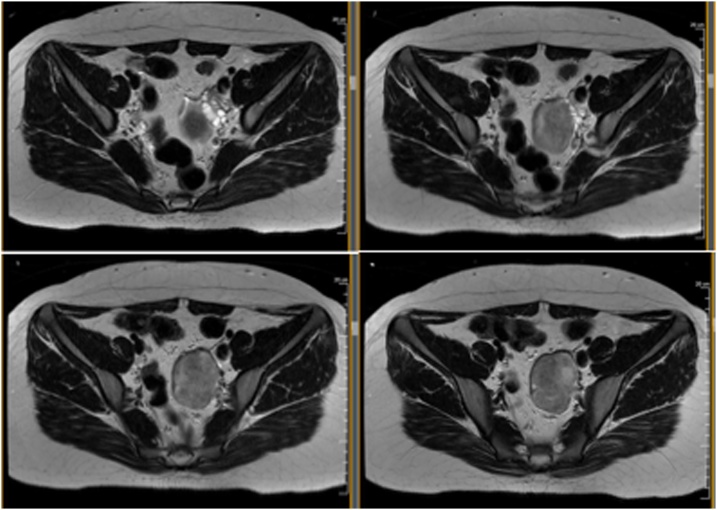
Clockwise from top left – cranial to caudal axial MRI imaging highlighting an 8-centimeter pelvic mass.

A 16-year-old girl with a history of irregular menses for one year presented to the surgical clinic after a screening ultrasound revealed a pelvic mass. She subsequently underwent a pelvic MRI which suggested that this 8-centimeter mass was distinct from the ovary and therefore the possibility of a paraganglioma or neuroblastic tumor was raised (Fig. 1). The patient denied any symptoms of flushing or palpitations, but did have occasional headaches. She was normotensive. Evaluation of plasma hormones revealed elevation of dopamine alone. Preoperative blockade was felt to be unnecessary and the patient underwent a laparoscopic-converted-to-open resection of the pelvic mass. In the operating room, a large retroperitoneal mass was identified just anterior to the sacrum. The majority of dissection was performed laparoscopically but the deepest aspect of the tumor, which contained its blood supply, could not be well-visualized. A small, lower midline incision was made in order to safely complete the resection of this deeper portion.

Postoperatively, the patient was monitored in the ICU. Overnight, she had severe anxiety and crying episodes requiring dexmedetomidine infusion, which was eventually converted to oral lorazepam. Her pain was well-controlled and she tolerated a regular diet on postoperative day one, but remained hospitalized for an additional two days predominantly to control her anxiety. By postoperative day three, the patient no longer required lorazepam and was able to be discharged home. In follow up, her mood had returned to baseline and she has had no evidence of recurrence in nearly two years. Pathologic evaluation confirmed the tumor to be a ganglioneuroma, maturing subtype.

## 3 Discussion

Ganglioneuromas are rare neural crest tumors that represent 6.4–20% of all neuroblastic tumors and have an overall reported incidence of 1 in 1,000,000 [1,[7], [8], [9]]. Many of these tumors are diagnosed when they grow large enough to affect surrounding structures, with the most common sites of origination being the adrenal medulla, organ of Zuckerkandl, or along the paravertebral sympathetic ganglia [1,9]. In one study, these tumors showed a predilection for growth within the thoracic cavity in 41.5% of cases, the abdominal cavity excluding adrenal origins 37.5% of the time, and 21% localized to the adrenal gland [5]. Depending on their location, they may present with an array of findings including, but not limited to abdominal pain, distention, constipation, dyspnea, paresthesias and claudication [1,10].

Few ganglioneuromas are hormonally active, a characteristic more often associated with neuroblastomas, pheochromocytomas, and paragangliomas [9]. Those that do exhibit metabolic activity usually secrete the catecholamines epinephrine and norepinephrine, either exclusively or in combination [11]. Intraoperative hypertensive crisis may occur when excess norepinephrine or epinephrine production is unrecognized or improperly managed pre-operatively [12]. Therefore, assessment of hormonal activity is essential to the management of these tumors and is accomplished via screening for serum or urinary catecholine metabolites including vanillylmandelic acid (VMA) and homovanillic acid (HVA) [13]. If a hormonally active tumor is identified, management includes first an alpha-blockade with phenoxybenzamine or phentolamine, followed by beta-blockers to help reduce tachycardia.

Ganglioneuromas can also secrete dopamine, either exclusively or in addition to epinephrine and norepinephrine. These are exceptionally rare as dopamine-secreting tumors are generally extra-adrenal pheochromocytomas and a review of the existing world literature highlights only 17 published cases and reports of dopamine producing ganglioneuromas to our knowledge [9,[14], [15], [16], [17], [18], [19], [20], [21], [22], [23], [24], [25], [26], [27], [28], [29]]. Of these, only 5 had exclusive secretion of dopamine or had excessive amounts of its inactive metabolite HVA, whereas the remainder were found to also secrete epinephrine and norepinephrines or had detectable levels of their inactive metabolite, VMA [14,[24], [25], [26],^29^].

This poses significant difficulty in appropriate diagnosis given the rarity of this type of tumor and the relative asymptomatic clinical presentation. When epinephrine and norepinephrine are present, the symptoms typically found include unexplained hypertension, facial flushing, palpitations, or diaphoresis [9,10]. However, in the case of dopamine, the patients are generally normotensive and have no systemic manifestations of excess hormone.

Imaging that may help distinguish between active and inactive neuroblastic masses includes the nuclear medicine study measuring metaiodobenzylguanidine (mIBG) uptake, which has increased uptake when catecholamines, HVA, or VMA are present. However, mIBG studies may further complicate the diagnosis as neuroblastomas and pheochromocytomas also have increased uptake of mIBG and uptake is not always present in ganglioneuromas [5]. Ultimately, the distinction between these tumors is made by pathologic examination. One study by Eisenhofer et al. proposed the use of plasma methoxytyramine and dopamine measurements rather than urine samples for the detection of tumors that produce exclusively dopamine, where urinary dopamine levels were non-specific and insensitive due to contributions from plasma DOPA and dependent on plasma dopamine in general [10]. This marker may help to identify tumors otherwise thought to be inactive when negative results are obtained from the standard screening tests.

Though dopamine-secreting ganglioneuromas may be clinically silent compared to those that secrete more vasoactive compounds, it remains important to appropriately diagnose a dopamine-secreting tumor. Dopamine exerts an antiadrenergic effect and prevents the vasoconstriction promoted by the other catecholamines, thereby limiting the hypertension that would other be present if there was a mixed-hormone secreting mass [30]. Removal of this inhibitory stimulus through pharmacological or surgical means may result in rebound hypertension from unopposed alpha and beta adrenergic activity, a complication to be aware of intraoperatively [9,14]. Additionally, dopamine is an important neurotransmitter; decreased dopamine levels are hypothesized to contribute to neurodegenerative and psychiatric disorders including Parkinson’s disease, depression, and mood-affective disorders [31]. While the effects of our patient’s post-operative emotional lability and depressive symptoms were transient, the acute decrease in dopamine stimulation after excision of the ganglioneuroma was likely the cause of her symptoms and has resolved.

Management of ganglioneuromas is traditionally limited to surgical excision without adjuvant or neoadjuvant chemoradiation [1,5,7,8]. Open or laparoscopic interventions are available as options and within the pediatric population the minimally-invasive route has become the therapeutic modality of choice [1]. However, although the excision procedure itself is low risk, surgical resection may be challenging in tumors that have grown significantly in size as they abut or encase major neurovascular structures or surrounding organs. While complete resection is ideal, incomplete excision is considered acceptable in situations where attempted resection could result in significant morbidity and mortality, as ganglioneuromas are associated with good prognoses [5,27]. In the cases of incomplete resection, the patient must be monitored closely for possible tumor progression, with particular concern if the residual tumor is 2 cm or greater as these are more likely to result in progression [5,7]. Additionally, for metabolically active ganglioneuromas, those that are completely excised show normalization of hormone levels after removal, but those that are not completely excised may be concerning for persistent elevation [9,[14], [15], [16], [17], [18], [19], [20], [21], [22], [23], [24], [25], [26], [27], [28], [29]]. Thus, follow up for these patients may include imaging studies, measurement of the levels of active compounds, and medical management.

Our patient represents the 6^th^ reported case to our knowledge of a ganglioneuroma that exclusively produced dopamine. She did not require pre-operative alpha or beta blockade and was managed with a laparoscopic-converted-to-open excision of the pelvic ganglioneuroma. Acute withdrawal of dopamine may have contributed to her emotional lability and depressive symptoms post-operatively. She is currently doing well and will be monitored closely for any recurrence.

## 4 Conclusions

Though uncommon, ganglioneuromas can be hormonally active and can secrete a variety of catecholamines that require appropriate peri-operative management prior to surgery. This complicates diagnosis as these neuroblastic tumors must be distinguished from pheochromocytomas, neuroblastomas, and paragangliomas. Therefore, appropriate work up including imaging and endocrine studies should be performed to determine management and prognosis. As our case study shows, diagnostic consideration should be made to specifically screen for dopamine secretion as isolated production is rare, but this stimulus may be attributable to post-resection psychiatric symptoms. Management can include incomplete surgical resection to reduce morbidity with close follow up and monitoring for progression.

## Funding

No study sponsors.

## Ethical approval

The case report has been approved for publication and reporting by the Institutional Review Board for our institution.

## Consent

Written informed consent was obtained from the patient for publication of this case report and accompanying images. A copy of the written consent is available for review by the Editor-in-Chief of this journal on request.

## Author contribution

Louis Chai: Investigation, resources, data curation, writing – original draft, writing – review and editing, visualization

Sean Ciullo: Conceptualization, investigation, resources, data curation, writing – original draft, writing – review and editing, visualization, supervision, funding acquisition. Pre- and post- operative care, operative surgeon.

Rajeev Prasad: Investigation, resources, conceptualization, data curation, writing – original draft, writing – review and editing, visualization, supervision, funding acquisition. Pre- and post-operative care, operative surgeon.

## Registration of research studies

Not a research study.

## Guarantor

Rajeev Prasad.

## Disclosures

The authors of this paper have no conflicts of interests or disclosures to be reported.

## Provenance and peer review

Not commissioned, externally peer-reviewed

## Declaration of Competing Interest

No conflicts of interests from any author.
